# Assessment of bilateral motor skills and visuospatial attention in children with perinatal stroke using a robotic object hitting task

**DOI:** 10.1186/s12984-020-0654-1

**Published:** 2020-02-13

**Authors:** Rachel L. Hawe, Andrea M. Kuczynski, Adam Kirton, Sean P. Dukelow

**Affiliations:** 1 Department of Clinical Neurosciences, Hotchkiss Brain Institute, University of Calgary, 3330 Hospital Drive NW, Calgary, AB T2N 4N1 Canada; 2 Cumming School of Medicine, University of Calgary, 3330 Hospital Drive NW, Calgary, AB T2N 4N1 Canada; 3 Department of Pediatrics, Alberta Children’s Hospital, 2888 Shaganappi Trail NW, Calgary, AB T3B 6A8 Canada; 4 Alberta Children’s Hospital Research Institute, Alberta Children’s Hospital, 2888 Shaganappi Trail NW, Calgary, AB T3B 6A8 Canada

**Keywords:** Cerebral palsy, Perinatal stroke, Robotics, Bilateral motor control, Visuospatial attention

## Abstract

**Background:**

While motor deficits are the hallmark of hemiparetic cerebral palsy, children may also experience impairments in visuospatial attention that interfere with participation in complex activities, including sports or driving. In this study, we used a robotic object hitting task to assess bilateral sensorimotor control and visuospatial skills in children with hemiparesis due to perinatal arterial ischemic stroke (AIS) or periventricular venous infarct (PVI). We hypothesized that performance would be impaired bilaterally and be related to motor behavior and clinical assessment of visuospatial attention.

**Methods:**

Forty-nine children with perinatal stroke and hemiparetic cerebral palsy and 155 typically developing (TD) children participated in the study. Participants performed a bilateral object hitting task using the KINARM Exoskeleton Robot, in which they used virtual paddles at their fingertips to hit balls that fell from the top of the screen with increasing speed and frequency over 2.3 min. We quantified performance across 13 parameters including number of balls hit with each hand, movement speed and area, biases between hands, and spatial biases. We determined normative ranges of performance accounting for age by fitting 95% prediction bands to the TD children. We compared parameters between TD, AIS, and PVI groups using ANCOVAs accounting for age effects. Lastly, we performed regression analysis between robotic and clinical measures.

**Results:**

The majority of children with perinatal stroke hit fewer balls with their affected arm compared to their typically developing peers. We also found deficits with the ipsilesional (“unaffected”) arm. Children with AIS had greater impairments than PVI. Despite hitting fewer balls, we only identified 18% of children as impaired in hand speed or movement area. Performance on the Behavioral Inattention Test accounted for 21–32% of the variance in number of balls hit with the unaffected hand.

**Conclusions:**

Children with perinatal stroke-induced hemiparetic cerebral palsy may have complex bilateral deficits reflecting a combination of impairments in motor skill and visuospatial attention. Clinical assessments and interventions should address the interplay between motor and visuospatial skills.

## Background

Perinatal stroke is the most common cause of hemiparetic cerebral palsy (HCP) [1]. Motor deficits have been well documented in the hemiparetic upper extremity, and include weakness [2–4], spasticity [5–7], and impaired selective motor control [8–10]. Additionally, many individuals have proprioceptive deficits [11–13]. Previous work has also established that deficits are not isolated to the contralesional side as impairments can often be found bilaterally [14–16]. While clinical assessments and research studies typically focus on reaching and grasping performance [17], real-world situations often require more complex actions. For instance, in a clinical assessment a child may be attempting to pick up a single block off a table, while in the real-world a child is trying to catch a fast-moving ball that may come from any direction within a field full of distractions. In contrast to the clinical assessment, this complex real-world activity requires not only preserved motor and sensory function bilaterally, but also the ability to use visuospatial skills to make rapid motor decisions. In order to better target treatments and measure change, assessments must address these complex skills.

Outside of sensorimotor impairments, HCP can impact a number of neural processes including visuospatial attention. Visuospatial attention refers to the cognitive function of attending to relevant environment stimuli while ignoring irrelevant stimuli, and consists of the processes of alerting, orienting, and executive control [18]. Deficits in visuospatial attention in children with HCP have been previously documented using primarily pencil and paper tests, with a recent study finding that over 60% of a sample of 75 children with HCP failed at least one test of visuospatial attention [19]. While visuospatial attention deficits are found in children with both right and left hemispheric damage, deficits may be more common and severe when the right hemisphere is affected [20, 21]. Right hemisphere damage is more commonly linked to left egocentric neglect (neglect of space to the left of body’s midline) [19], whereas the deficits with left hemisphere lesions may be more bilateral [19–21] and exacerbated by more complex tasks [20]. In an attention task involving listening, both right and left hemisphere lesions had deficits in shifting attention between sides [22]. The type of lesion may also impact visuospatial attention deficits, with deficits being more prevalent in children with cortical/subcortical lesions (arterial stroke) compared to periventricular (venous) lesions, possibly due to the regions damaged [19]. While visuospatial attention is a known prerequisite to movement, the interplay between deficits in attention and motor impairments in HCP is not well understood.

Robotic tasks have the advantage of being able to integrate visuospatial attention demands with motor tasks in a controlled manner to precisely monitor participants’ performance. A robotic object hitting task has been previously developed and applied to adults with stroke to assess bilateral motor performance and visuospatial attention, and found to correlate with the Functional Independence Measure and Behavioral Inattention Test [23]. In this task, participants use virtual paddles on the ends of both hands to hit away virtual balls that fall with increasing speed and frequency. Good performance on the task, as measured by hitting as many balls as possible, therefore requires integration of visuospatial attention and motor skills.

In this study, we employed a robotic object hitting task to quantify performance in children with hemiparetic cerebral palsy due to either a perinatal arterial ischemic stroke (AIS) or periventricular venous infarct (PVI) compared to typically developing (TD) children. We examined how success on the task (i.e. number of balls hit) related to motor behavior during the task, namely hand speed and movement area, as well as to performance on a more simplistic visually guided reaching task. We also looked at the relationship between clinical measures of both motor and visuospatial attention with performance on the object hitting task. We hypothesized that performance on the task would be impaired bilaterally in children with HCP, and correlate with both motor behavior and clinical assessment of visuospatial neglect.

## Methods

### Participants

We recruited participants with hemiparesis due to perinatal stroke to participate in this study from a population-based cohort (the Alberta Perinatal Stroke Project) [24]. Inclusion criteria were: 1) age 6 to 19 years old; 2) MRI confirmation of unilateral perinatal stroke (AIS or PVI); 3) clinical confirmation of symptomatic hemiparesis; 4) gestational age > 36 weeks; 5) visual acuity of at least 20/50; and 6) written informed consent/assent. Exclusion criteria were: 1) multifocal stroke; 2) other neurological disorders not attributable to perinatal stroke; 3) Manual Abilities Classification System grade V; 4) severe spasticity demonstrated by Modified Ashworth Scale > 3 in the upper extremity; 5) inability to comply with the study protocol; 6) upper extremity surgery, botulinum toxin treatment, constraint induced movement therapy, or brain stimulation therapy within 6 months of study participation.

Typically developing (TD) children between the ages of 6 and 19 with no history of neurologic impairment also completed the same robotic assessments as the participants with hemiparesis. All provided written informed assent or consent. The University of Calgary Conjoint Health Research Ethics Board approved this study.

### Robotic assessments

**Fig. 1:**
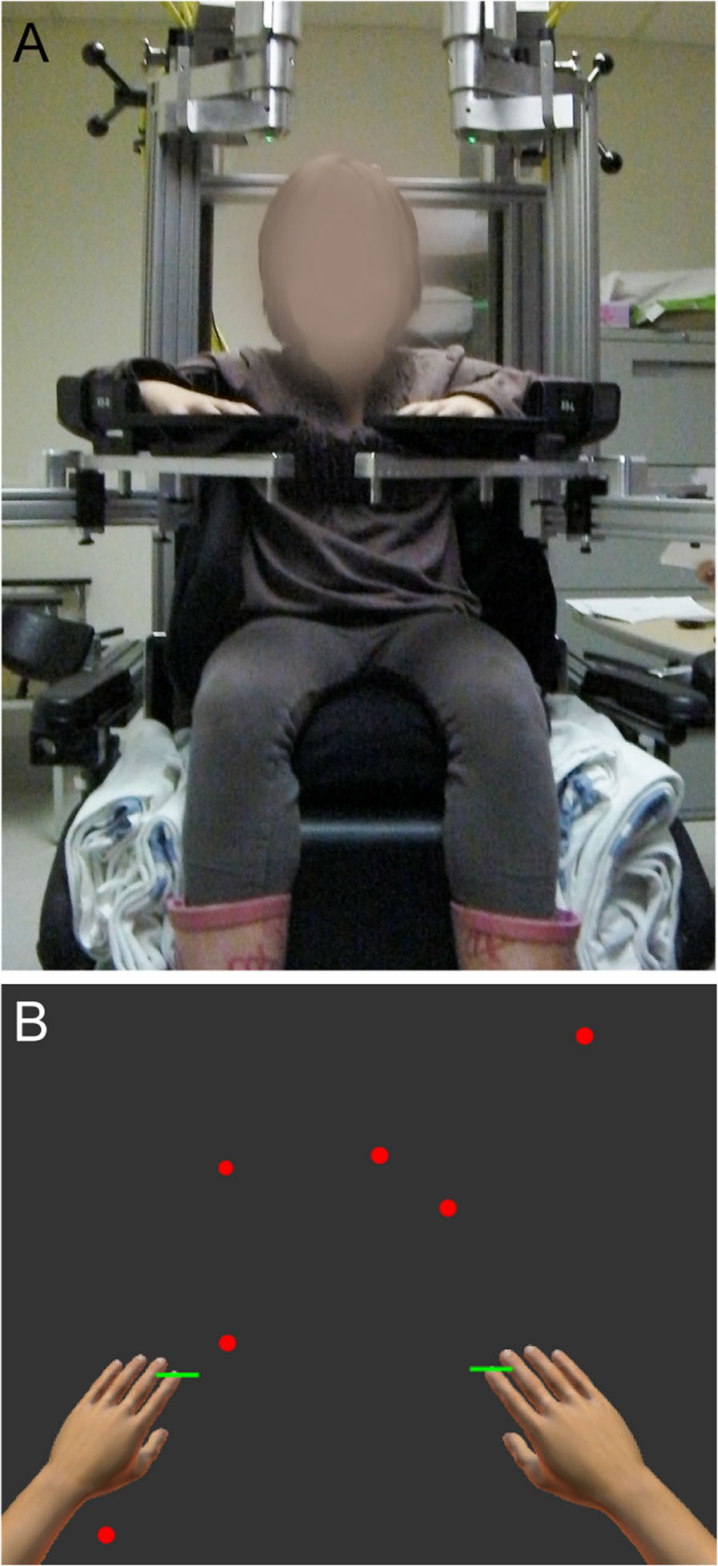
KINARM exoskeleton and Object Hit task. **a** KINARM exoskeleton is shown with wheelchair base and arm troughs that support arms in horizontal plane. We used adaptations including booster seats and smaller arm troughs as needed to accommodate smaller children. **b** Depiction of the task workspace, with green virtual paddles displayed at the participant’s fingertips. Balls fell from the top of the screen from 10 different horizontal locations

Participants completed standardized robotic assessments using the KINARM robotic exoskeleton (Kinarm, Kingston, Ontario) as shown in Fig. 1a. Participants sat in a wheelchair base modified to accommodate pediatric participants. Adjustable troughs supported the arms in the horizontal plane. The robot allowed for free elbow and shoulder movement in the horizontal plane. A horizontal display provided augmented reality.

### Object hit task

For the object hitting task, children used virtual paddles displayed at their fingertips. Balls moved towards the child from the top of the screen from one of 10 horizontal locations or bins. We instructed the children to hit as many balls as possible with either hand. The workspace is depicted in Fig. 1b. The robot delivered a small haptic force pulse when the ball contacted the paddle. The magnitude of the force varied based on the ball’s acceleration following the hit, however in general was less than 5 N. Balls fell with increased speed and frequency throughout the 2 min and 18 s of the task, for a total of 300 balls. The object hitting task in adults has been previously described in detail [23].

We quantified performance on the task using 13 parameters described below [23, 25]. We refer to individuals with perinatal stroke as having an “affected” (hemiparetic) arm and an “unaffected” arm based on their clinical assessments. We acknowledge that while we are using the terms “affected” and “unaffected” for brevity, motor deficits have been found bilaterally in children with hemiparesis [14, 15]. For TD participants, we operationally use the terms “affected” as analogous to “non-dominant” and “unaffected” to “dominant”.

*Overall Performance Parameters:*

1. Total Hits: Total number of balls hit by either hand out of maximum of 300 balls
2. Hits Affected: Number of balls hit by the affected hand
3. Hits Unaffected: Number of balls hit by unaffected hand

*Spatial and Temporal Performance Parameters:*

4) Miss Bias: Quantifies spatial bias in misses towards one side of the workspace or the other. A weighted mean is calculated (shown below) based on the number of misses in each of the 10 bins along the x-axis that balls fall from and their position along the x-axis. To compare right and left affected sides, the we flipped the workspace for participants who were left-side affected, thereby positive values indicate more misses toward the side of the affected or non-dominant arm.
  $$ miss\ bias=\frac{\sum_{i=1}^{10}{x}_i\bullet {w}_{m,i}}{\sum_{i=1}^{10}{w}_{m,i}} $$

where i refers to the bin (horizontal location), x_i_ refers to the x location of the i^th^ bin and weight w_m, i_ is the number of misses in the i^th^ bin.

5) Hand Transition: Describes the location in the workspace where hand preference switches. This parameter uses a subset of bins consisting of bins where both hands made hits, as well as one additional bin on each side of bins with overlapping hits. Weighted means of hit distributions for each hand are calculated for this subset of bins. The average of the weighted means for each hand is then calculated to determine the location of the hand transition, as shown below:
  $$ hand\ transition=\left(\frac{\sum_{i=1}^{10}{x}_i\bullet {w}_{R,i}}{\sum_{i=1}^{10}{w}_{R,i}}+\frac{\sum_{i=1}^{10}{x}_i\bullet {w}_{L,i}}{\sum_{i=1}^{10}{w}_{L,i}}\right)/2 $$

x_i_ refers to the x-location of the i^th^ bin and weight w_R(L), i_ refers to number of hits with the right (or left) hand in the i^th^ bin. If the i^th^ bin was not part of the specified subset, the weight was set to zero. If there were no bins with hits from both the right and left hands, the subset of bins was defined to include the furthest right bin in which hits were made with the left hand and furthest left bin in which hits were made with the right hand.

As with miss bias, we flipped the workspace for participants who were left-side affected, so positive values always indicate the transition was shifted toward the affected arm.

6) Median Error: The percentage of the way through the task (based on number of balls) when 50% of the total misses occurred. For example, if a participant misses 80 balls during the task, the median error is the ball number at which the 40th miss occurred, divided by 300 (total balls). A higher median error indicates that most errors occurred during the later portion of the task when it was more difficult.

*Motor Performance Parameters:*

7) Hand Speed Affected: Mean hand speed of the affected hand over the entire duration of the task (m/s).
8) Hand Speed Unaffected: Mean hand speed of the unaffected hand over the entire duration of the task (m/s).
9) Movement Area Affected: Area of space covered by the movements of the affected hand during the task, as determined by defining a convex hull (polygon) that includes the boundaries of the movement trajectory of the affected hand (m^2^).
10) Movement Area Unaffected: Area of space covered by the movements of the unaffected hand during the task, as determined by defining a convex hull (polygon) that includes the boundaries of the movement trajectory of the unaffected hand (m^2^).

*Interlimb Asymmetry Parameters:*

11) Hand Bias of Hits: Quantifies asymmetry in hits between hands, calculated as (Hits Unaffected – Hits Affected)/(Hits Unaffected + Hits Affected).
12) Hand Speed Bias: Quantifies asymmetry in hand speed between hands, calculated as (Hand Speed Unaffected – Hand Speed Affected)/(Hand Speed Unaffected + Hand Speed Affected)
13) Movement Area Bias: Quantifies asymmetry in movement area between hands, calculated as (Movement Area Unaffected – Movement Area Affected)/(Movement Area Unaffected + Movement Area Affected)

### Visually guided reaching task

To understand the relationship between performance on the bimanual object hit task with a simpler motor task, we also analyzed performance on a visually guided reaching task. The full results of the of this task in the same cohort have been previously reported [14]. In brief, children performed center out reaches to one of 4 targets. For this analysis, we used the following parameters [26]:

1. Reaction Time: Time from target appearing to onset of arm movement (s)
2. Initial Direction Error: Angular deviation between the straight-line path from center to peripheral target and the vector from the hand position at movement onset to completion of initial movement (degrees)
3. Path Length Ratio: Ratio of total distance hand traveled from movement onset to offset to minimum distance between center and peripheral target
4. Maximum Speed: Maximum hand speed between movement onset and offset (cm/s)

We selected these parameters to represent temporal (reaction time and maximum speed) and spatial (initial direction error and path length ratio) components of movement that were hypothesized a priori to impact performance on the object hit task.

### Clinical assessments

Participants with perinatal stroke completed a battery of clinical assessments administered and scored by a trained pediatric occupational therapist.

1. Assisting Hand Assessment: Assesses how effective a child is at using their affected arm to complete bimanual tasks, with focus on typical performance not capacity. Scores are transformed to a logit-scale ranging from 0 (no use of affected arm) to 100 (full use of affected arm) [27].
2. Melbourne Assessment of Unilateral Upper Limb Function: Assesses reaching and grasping function with scores ranging from 0 (worst) to 100 (best) [28].
3. Behavioral Inattention Test: The conventional subset of tests includes the following 6 pencil and paper tests of visuospatial neglect: line, star, and letter cancellation, line bisection, and figure copying and drawing. The maximum score is 146, with scores below 130 being indicative of visuospatial neglect [29].

### Statistical analysis

To account for expected age-related changes [30], we determined age-predicted norms. For each parameter, we fit a second-order polynomial curve to the TD children’s performance across the age range and calculated 95% prediction interval bands. We could then identify the number of children with AIS and PVI that fell outside of the age-predicted norms. This allowed us to detect impairments on an individual level rather than relying on group differences. In addition to identifying the number of children with impairments, we compared performance on each parameter between TD children, children with AIS, and children with PVI using one-way ANCOVAs with age as a covariate. When we found significant between-group differences, we conducted post-hoc comparisons between groups using Bonferroni corrections for multiple comparisons (Bonferroni corrected *p*-value = 0.05/3 = 0.017). To determine the impact of side of hemiparesis, we compared individuals with right arm affected to those with left arm affected hemiparesis using ANCOVAs with age as a covariate. We completed regression analysis between hits with each hand and hand speed and movement area during the OH task, as well as between hits with each hand and selected visually guided reaching parameters and clinical assessments. We used SPSS 24 (IBM Corp, Armonk, NY) and Matlab 2015a (Mathworks, Nantick, MA) for statistical analyses.

## Results

### Participants

Table 1 shows demographic and clinical characteristics of participants. A total of 155 typically developing children and 49 children with perinatal stroke (28 AIS, 21 PVI) completed the study. There was no significant difference in age between the three groups (F = 0.368, *p* = 0.692).

**Table 1:** Demographic and Clinical Characteristics

	TD	AIS	PVI
*N*	155	28	21
*Male/Female*	81/74	18/10	15/6
*Affected Arm*	Handedness: 1 A/13 L/141 R	9 L/19 R	11 L/10 R
*Age (mean years ± SD)*	12.5 ± 4.0	12.4 ± 4.0	11.7 ± 3.8
*MACS*		MACS I: *N* = 4, MACS II: *N* = 16	MACS I: *N* = 8, MACS II: *N* = 5
*BIT (mean ± SD)*		129 ± 20.9	139.0 ± 5.5
		range: 56–145	range: 122–146
		*N* = 6 below cutoff for neglect (< 130)	N = 1 below cutoff for neglect (< 130)
*AHA (mean ± SD)*		61.3 ± 20.5	75.2 ± 16.7
		range: 32–100	range: 55–100
*Melbourne (mean ± SD)*		69.1 ± 20.8	89.4 ± 11.1
		range: 31–100	range: 64–100

*MACS* Manual Ability Classification Scale, *BIT* Behavioral Inattention Test, *AHA* Assisting Hand Assessment. MACS, AHA, and Melbourne scores were unavailable for 16 children (8 AIS, 8 PVI), and BIT scores were unavailable for 2 children (1 AIS, 1 PVI)

### Age effects on performance on robotic object hit task

**Fig. 2:**
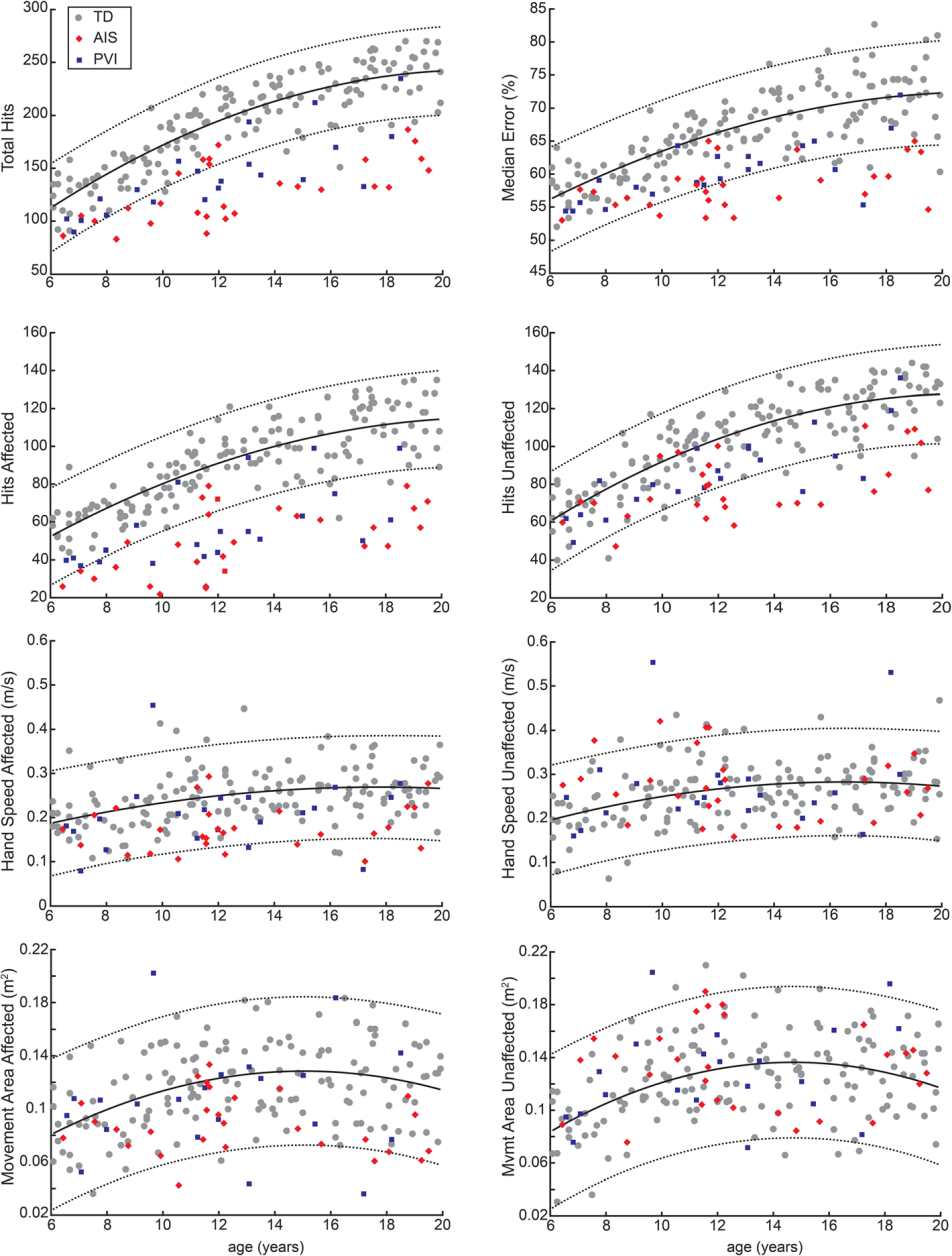
Age Curves. Age curves (solid black line) and 95% prediction bands (dotted lines) were calculated based on TD participants (grey circles). AIS participants are shown by the red diamonds, and PVI participants by the blue squares

We found significant effects of age in TD children for total hits, median error, hits affected, hits unaffected, hand speed affected, hand speed unaffected, movement area affected, and movement area unaffected. Figure 2 shows the age curves based on TD participants with 95% prediction bands, with AIS and PVI participants superimposed.

### Effect of affected side

Comparisons between children with right arm affected and left arm affected hemiparesis did not show any significant differences for any of the parameters, except for movement area bias (F = 4.99, *p* = 0.030), where children whose left arm is affected had a greater movement area bias compared to children whose right arm is affected.

### Percentage of children with impairments on each parameter

**Fig. 3:**
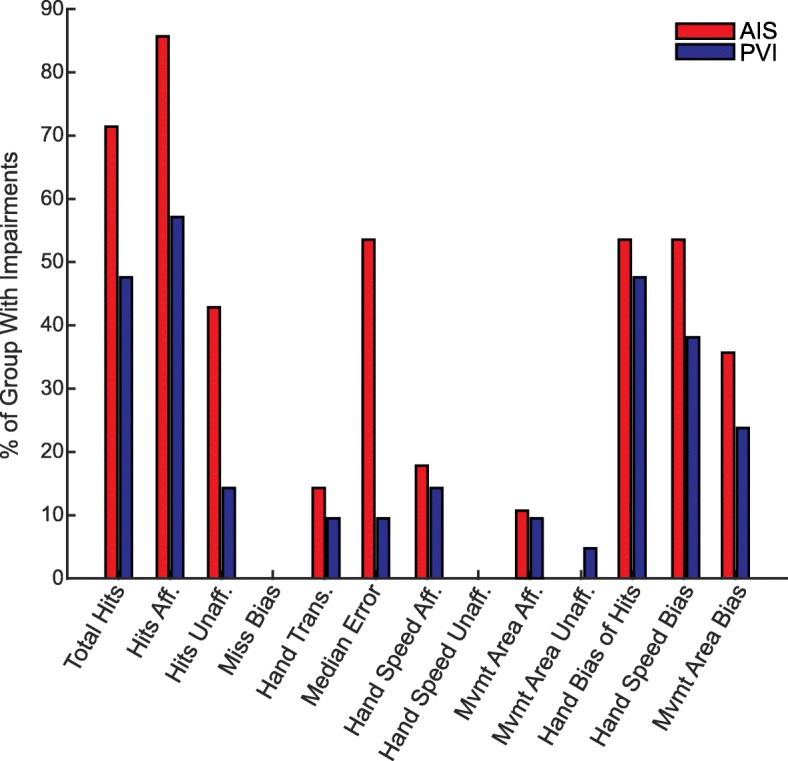
Percentage of Children with Impairments*.* Children who fell outside the 95% prediction bands were determined to be impaired. For each parameter, the percentage of the AIS (red) and PVI (blue) groups with impairments are shown

Figure 3 shows the percentage of children with impairments on each parameter as identified by the 95% prediction bands. Overall, we identified a higher proportion of children with AIS as impaired compared with PVI. Total hits and hits with the affected hand had the greatest percentage of impaired participants, notably with over 80% of the AIS group impaired on hits with affected hand. Hits with the unaffected hand was impaired in 43% of AIS and 14% of PVI. We identified fewer children as having impairments in the motor performance parameters (hand speed and movement area) for either hand, however, the bias between the hands for both parameters did identify a significant portion of children as impaired. Interestingly, we did not identify any children as having impairments in miss bias, indicating misses were not systematically occurring on one side of the workspace.

### Group differences

**Fig. 4:**
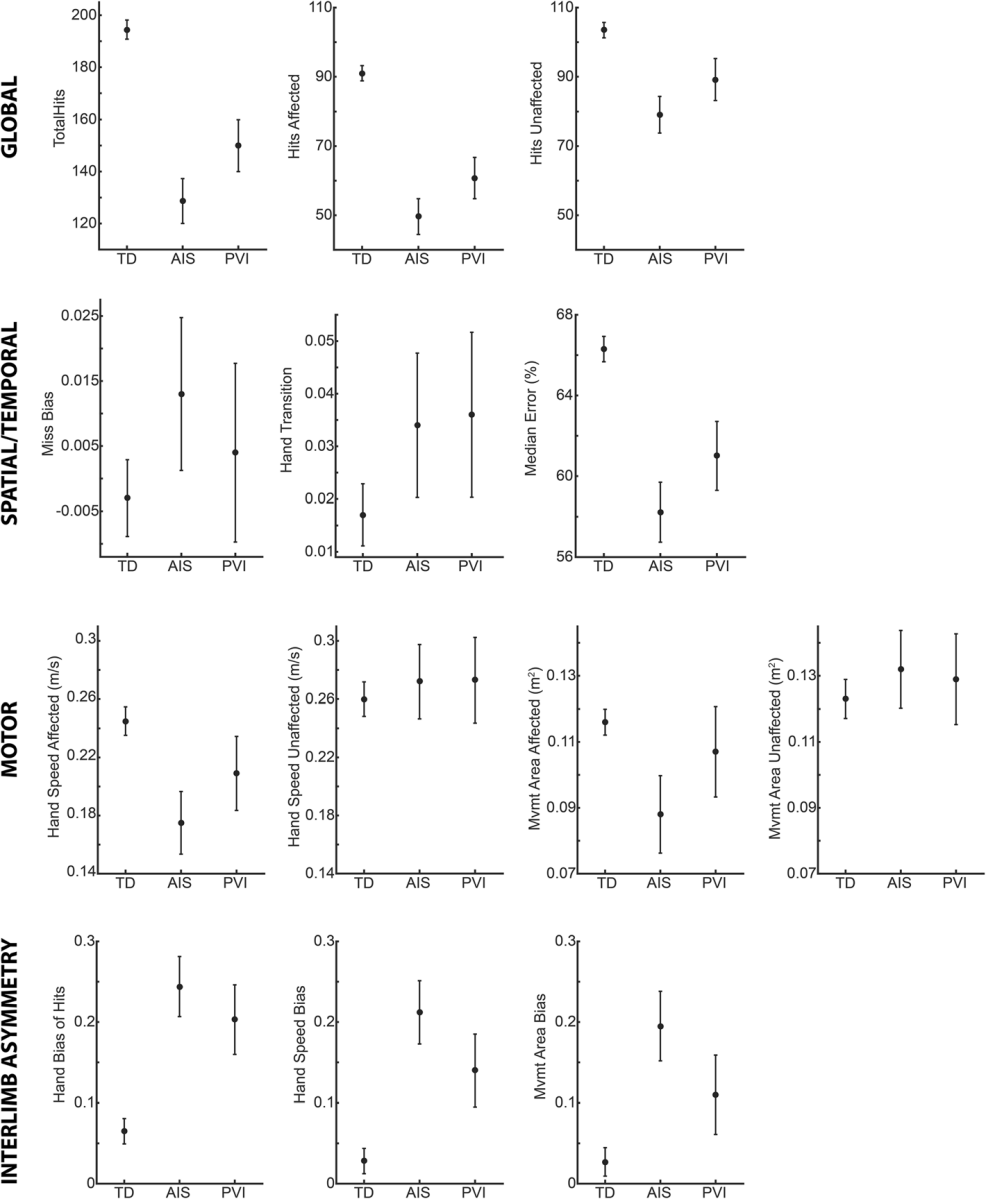
Group Means. Age-adjusted group means and 95% confidence intervals are shown for each parameter

Figure 4 shows age-adjusted group means and 95% confidence intervals for TD, AIS, and PVI groups for each parameter. Between-group differences are described below:

#### Overall Performance

We found significant between group differences for total hits (F = 115.3, *p* < 0.001), hits affected (F = 131.7, *p* < 0.001), and hits unaffected (F = 40.4, p < 0.001). For total hits, AIS and PVI both had fewer hits than TD (TD vs. AIS: t = 13.8, p < 0.001; TD vs. PVI: t = 8.2, *p* < 0.001), and AIS had fewer hits than PVI (t = − 3.2, *p* = 0.003). Hits with the affected hand showed the same pattern, with both AIS and PVI having fewer hits than TD (TD vs. AIS: t = 14.4, *p* < 0.001, TD vs. PVI: t = 9.3, p < 0.001) and AIS having fewer hits than PVI (t = − 2.8, *p* = 0.008). For hits with the unaffected hand, AIS and PVI again had fewer hits than TD (TD vs. AIS: t = 8.4, *p* < 0.001, TD vs. PVI: t = 4.3, p < 0.001), but no difference was found between AIS and PVI: t = − 2.5, *p* = 0.017).

#### Spatial/Temporal Performance

We did not find a between group difference for miss bias. For hand transition, we found a significant between group effect (F = 4.554, *p* = 0.012), however, no post-hoc tests were significant. Median error showed between group differences (F = 58.4, *p* < 0.001), with post-hoc tests showing median error was reduced (errors occurring earlier in the task) in AIS and PVI compared to TD (TD vs. AIS: t = 9.9, *p* < 0.001; TD vs. PVI: t = 5.7, p < 0.001). AIS was not significantly different than PVI (t = − 2.4, *p* = 0.020).

#### Motor Performance

There was a significant between group difference for hand speed of the affected hand (F = 17.2, *p* < 0.001), with both AIS and PVI having decreased hand speed compared to TD (TD vs. AIS: t = 5.8, p < 0.001; TD vs. PVI: t = 2.6, *p* = 0.010), however, there was no difference between AIS and PVI (t = − 2.0, *p* = 0.051). Movement area of the affected hand was also significantly different between groups (F = 10.4, *p* < 0.001), however, only AIS was significantly smaller than TD (t = 4.4, *p* < 0.001). We did not find any between group differences for hand speed (F = 0.635, *p* = 0.531) or movement area (F = 1.247, *p* = 0.290) of the unaffected hand.

#### Interlimb Asymmetry

We found a significant between group difference for hand bias of hits (F = 48.2, *p* < 0.001), with both AIS and PVI having a bias of more hits with the unaffected hand compared to TD (TD vs. AIS: t = − 8.7, *p* < 0.001; TD vs. PVI: t = − 5.9, *p* < 0.001), though no difference in bias between AIS and PVI (t = 1.4, *p* = 0.165). Hand speed bias had significant differences between groups (F = 42.2, *p* < 0.001), with AIS and PVI showing greater bias compared to TD (TD vs. AIS: t = − 8.5, p < 0.001; TD vs. PVI: t = − 4.6, p < 0.001) but no difference between AIS and PVI (t = 2.4, *p* = 0.022). Movement Area Bias also had significant between group differences (F = 27.0, p < 0.001), with AIS and PVI showing greater bias compared to TD (TD vs. AIS: t = − 7.1, p < 0.001; TD vs PVI: t = − 3.1, *p* = 0.002), and AIS had greater bias than PVI (t = 2.55, *p* = 0.014).

### Relationship between motor behavior and hits during the object hit task

We performed regressions between motor performance parameters (hand speed and movement area) and number of hits with each hand to determine the extent to which the amount and speed of movement can explain successful task performance (for example, is a child not hitting balls because they simply are not moving their affected arm during the task?). Table 2 shows the results of the regression analyses (R^2^ and *p*-values). Within the object hit task, the hand speed and movement area accounted for more of the variance in hits with either hand for the TD group compared to children with hemiparesis.

**Table 2:** Regressions Between Hits with Each Hand and Clinical Measures

	TD	AIS	PVI	AIS + PVI
*AFFECTED HAND*				
OH- Hand Speed	R^2^ = 0.31, *p* < 0.001	R^2^ = 0.13, *p* = 0.06	R^2^ = 0.07, *p* = 0.26	R^2^ = 0.11, *p* = 0.02
OH- Movement Area	R^2^ = 0.27, *p* < 0.001	R^2^ = 0.02, *p* = 0.46	R^2^ = 0.05, *p* = 0.34	R^2^ = 0.05, *p* = 0.11
VGR-Reaction Time	R^2^ = 0.46, *p* < 0.001	R^2^ = 0.34, *p* = 0.002	R^2^ = 0.33, *p* = 0.007	R^2^ = 0.32, *p* < 0.001
VGR-Initial Direction Error	R^2^ = 0.33, *p* < 0.001	R^2^ = 0.31, *p* = 0.003	R^2^ = 0.11, *p* = 0.13	R^2^ = 0.23, *p* < 0.001
VGR-Path Length Ratio	R^2^ = 0.27, *p* < 0.001	R^2^ = 0.28, *p* = 0.005	R^2^ = 0.03, *p* = 0.42	R^2^ = 0.16, *p* = 0.005
VGR-Maximum Speed	R^2^ = 0.01, *p* = 0.14	R^2^ = 0.005, *p* = 0.74	R^2^ = 0.13, p = 0.11	R^2^ = 0.07, *p* = 0.08
BIT	n/a	R^2^ = 0.18, *p* = 0.03	R^2^ = 0.11, *p* = 0.16	R^2^ = 0.14, *p* = 0.01
Melbourne	n/a	R^2^ = 0.49, *p* < 0.001	R^2^ = 0.02, *p* = 0.64	R^2^ = 0.31, *p* < 0.001
AHA	n/a	R^2^ = 0.47, *p* < 0.001	R^2^ = 0.05, *p* = 0.47	R^2^ = 0.30, *p* < 0.001
*UNAFFECTED HAND*				
OH- Hand Speed	R^2^ = 0.32, *p* < 0.001	R^2^ = 0.05, *p* = 0.24	R^2^ = 0.15, *p* = 0.09	R^2^ = 0.09, *p* = 0.03
OH- Movement Area	R^2^ = 0.24, *p* < 0.001	R^2^ = 0.07, *p* = 0.16	R^2^ = 0.15, p = 0.08	R^2^ = 0.10, *p* = 0.029
VGR-Reaction Time	R^2^ = 0.43, *p* < 0.001	R^2^ = 0.23, *p* = 0.01	R^2^ = 0.46, *p* < 0.001	R^2^ = 0.32, *p* < 0.001
VGR-Initial Direction Error	R^2^ = 0.23, *p* < 0.001	R^2^ = 0.005, *p* = 0.73	R^2^ = 0.40, *p* = 0.002	R^2^ = 0.08, *p* = 0.057
VGR-Path Length Ratio	R^2^ = 0.26, *p* < 0.001	R^2^ = 0.09, *p* = 0.13	R^2^ = 0.47, *p* < 0.001	R^2^ = 0.21, *p* < 0.001
VGR-Maximum Speed	R^2^ = 0.02, p = 0.09	R^2^ = 0.02, *p* = 0.46	R^2^ = 0.08, *p* = 0.21	R^2^ = 0.08, *p* = 0.054
BIT	n/a	R^2^ = 0.21, *p* = 0.01	R^2^ = 0.32, *p* = .009	R^2^ = 0.18, *p* = 0.003
Melbourne	n/a	R^2^ = 0.13, *p* = 0.12	R^2^ = 0.01, *p* = 0.70	R^2^ = 0.08, *p* = 0.11
AHA	n/a	R^2^ = 0.11, *p* = 0.15	R^2^ = 0.002, *p* = 0.88	R^2^ = 0.07, *p* = 0.15

R^2^ and p-values are shown for all regression analyses. AFFECTED HAND (top) refers to correlations between hits with the affected hand and Object Hit (OH) and Visually Guided Reaching (VGR) parameters of the affected hand as well as clinical measures. UNAFFECTED HAND (bottom) refers to correlations between hits with the unaffected hand and OH and VGR parameters for this hand as well as clinical measures. Note that the Melbourne Assessment only assessed the affected hand, so this is used in correlations with both hits of the affected and unaffected hands

### Relationship between object hit and visually guided reaching

When regressing the number of hits with selected parameters from the visually guided reaching task, we found reaction time explained the most variance in number of hits for either hand in the children with hemiparesis as well as the TD children. Initial direction error and path length ratio were related to hits on the affected hand for the TD and AIS groups, and with the unaffected hand for the TD and PVI groups. We did not find maximum speed in the visually guided reaching task to be related to hits for any group.

### Relationship between object hit and clinical assessments

Regressions between clinical assessment scores and hits found that BIT scores accounted for 21–32% of the variance in hits with the unaffected hand, a greater contribution than found for the affected hand. The Melbourne and AHA accounted for over 47% of the variance in hits with the affected hand for AIS, but did not significantly relate to hits for the PVI group or hits with the unaffected hand for either group.

## Discussion

The aim of this study was to examine the ability of children with perinatal stroke and hemiparetic cerebral palsy to perform a bilateral object hitting task. This task requires the integration of both motor and visuospatial attention skills and provides insights beyond a typical reaching task. We found that the majority of children with hemiparetic cerebral palsy had deficits in their ability to hit the targets, despite only a minority having deficits in hand speed or movement area during the task. We found that children with AIS had greater impairments than those with PVI. The difficulty children had in hitting the targets is likely a combination of bilateral motor impairments and visuospatial attention deficits.

While not the only factor, preserved motor function in the affected arm is a key component for success (hitting more targets) on the task. The majority (86% of AIS and 57% of PVI) of children with HCP hit fewer targets with their affected hand compared to their typically developing peers. A simple explanation for hitting fewer targets might have been that these children moved their hands with a slower speed or over a smaller area. However, we found that only a small fraction of the children with AIS or PVI had impairments in hand speed or movement area. Additionally, in TD children we found faster hand speed and larger movement area was related to hitting more targets, however, these relationships were not found in children with HCP. Regression analysis between select parameters from the visually guided reaching task and number of targets hit with the affected arm can give further insight into the extent to which motor impairments can impact performance on this task. Reaction time, which has both motor and attentional components, correlated with hits in TD as well as AIS and PVI, though to a slightly lesser degree than TD. It is likely that reaction time reflects an individual’s ability to rapidly attend to a new visual stimulus and form a motor plan, both of which are required to successfully hit the targets in the object hit task. Initial direction error and path length ratio, both of which relate to accuracy, explained 26–32% of the variance in targets hit for TD and AIS. However, these measures of accuracy were not correlated with hits for PVI. These findings suggest that motor deficits are not the only factor impacting a child’s performance on the object hitting task.

The object hit task also identified impairments in the unaffected (ipsilesional) arm, with children with HCP hitting fewer targets with this arm compared to TD children. As with the affected arm, we did not find deficits in movement speed or area on the task in the unaffected arm. Deficits in the unaffected arm have been reported previously, and have been hypothesized to reflect reorganization of the motor areas to the contralesional hemisphere [14–16]. While children may demonstrate compensatory strategies with their unaffected arm in their daily life, such as tying their shoes or dressing with one hand, only a small minority of children with hemiparetic cerebral palsy demonstrated compensatory increases in hand speed (14% of AIS, 9.5% of PVI) or movement area (3.6% of AIS, 9.5% of PVI) with their unaffected arm that exceeded the normative ranges. As motor impairments are typically more subtle in the unaffected arm, it is likely that deficits in visuospatial attention also impacted the ability of children to hit the targets with the less affected arm. This is evident in finding the Behavioral Inattention Test scores explained 21–32% of the variance in the number of hits with the unaffected arm. An additional cause of deficits in the unaffected arm may be due to the child having to attend to the affected arm to the detriment of the unaffected arm. Previous work has shown that children must visually monitor their affected limb more closely to compensate for sensorimotor deficits, which may in turn detract attention from the unaffected arm [31, 32].

The visuospatial attention demands of the object hit task are greater than those of simpler visually guided reaching tasks or common clinical assessments such as the Melbourne Assessment. Deficits in visuospatial attention may be the reason children hit fewer targets despite having largely normal magnitudes of movement during the task and may be especially true for the less affected arm. The widespread deficits found are consistent with previous findings on the prevalence of visuospatial attention deficits in hemiparetic cerebral palsy [19]. Our study is unique in that it assessed visuospatial attention in the context of a motor task, whereas most studies use traditional pencil and paper assessments, such as line bisection or star cancellation. Unlike these assessments where time is not a factor, the object hit task required rapid motor actions, as well as quickly shifting attention or dividing attention between sides of the workspace. These aspects of the object hit task may make it more translatable to real world situations such as sports or driving. The task also exposed how deficits may impact each other. For instance, the increased attention needed to make movements with the affected arm may have shifted attention away from the rest of the workspace, which typical assessments of visuospatial attention would not detect. Previous studies have also reported deficits in anticipatory gaze control [33], which would then cause movements to occur with a delay [34], making reaches towards moving targets ineffectual.

Our findings are consistent with the previous limited evidence on visuospatial attention in HCP showing that, unlike in adult stroke, deficits are not primarily associated with right parietal or frontal lesions, nor manifested by clear hemispatial neglect [19–21]. The object hit task has been used in adult stroke and offers an interesting contrast to our results in HCP [23]. In the adult stroke population, a clear pattern emerges of individuals with hemispatial neglect having greater impairments than those without. A key parameter in adult stroke is “Miss Bias”, where individuals with left hemispatial neglect demonstrate a significant bias in missing more targets toward the left side of the workspace. In contrast, we did not identify a single child in our study as impaired on this parameter, supporting previous findings that visuospatial attention deficits in HCP may not be manifested as clear unilateral neglect [19–21]. Additionally, we did not find differences in performance between children with right and left sided lesions except on one parameter (movement area bias), which is consistent with prior studies [19–21]. The differences in visuospatial attention deficits between perinatal and adult strokes may be due to the perinatal strokes occurring prior to the lateralization of attention [35, 36]. While deficits in children may be due to the lesion itself, they may also be influenced by differences in the acquisition of motor skills, as visuospatial attention has been shown to develop with locomotor experience in early childhood [37]. Development with asymmetrical use of their limbs may impact visuospatial attention, as well as decreased participation in activities such as sports compared to TD peers.

We found that the type of lesion had an effect of the severity of the deficits found, with AIS having more severe and widespread impairments compared to PVI. This supports prior findings of differences between lesion types in terms of motor impairments [14, 38] and visuospatial attention [19]. The increased severity in AIS lesions may be due to larger lesions [39] that are directly impacting frontal, parietal, and temporal cortical regions implicated in attention [40–42]. The differences between AIS and PVI could also be due to reorganization patterns. As AIS lesions occur later in development compared to PVI, the ability of cortical areas to reorganize may be decreased [43–45]. Additionally, the more significant motor impairments in AIS may impact the activity-driven development of visuospatial attention more than the milder impairments seen in PVI.

## Conclusions

Our study demonstrated that the majority of children with HCP had deficits in a bimanual object hitting task. These deficits are likely due to impairments in both motor skills and visuospatial attention. Our findings are important to rehabilitation of children with HCP, as typical clinical assessments do not have significant visuospatial attention demands, despite the importance of visuospatial attention on real-world tasks. As visuospatial attention impacts motor skills bilaterally, we recommend incorporating assessments of visuospatial attention, such as the Behavioral Inattention Test, into clinical and research assessments, though we acknowledge this assessment has limitations. We also suggest that rehabilitation programs for children with HCP should incorporate visuospatial attention. While this study demonstrated the interplay between bilateral motor skills and visuospatial attention, future work incorporating eye tracking can increase our understanding and potentially lead to targeted interventions.

## Data Availability

The datasets analyzed during the current study are available from the corresponding author on reasonable request.

## Abbreviations

AHA: Assisting Hand Assessment
AIS: Arterial Ischemic Stroke
BIT: Behavioral Inattention Test
HCP: Hemiparetic Cerebral Palsy
OH: Object Hit
PVI: Periventricular Venous Infarction
TD: Typically Developing
VGR: Visually Guided Reaching
